# Analysis of nuclear and organellar genomes of *Plasmodium knowlesi* in humans reveals ancient population structure and recent recombination among host-specific subpopulations

**DOI:** 10.1371/journal.pgen.1007008

**Published:** 2017-09-18

**Authors:** Ernest Diez Benavente, Paola Florez de Sessions, Robert W. Moon, Anthony A. Holder, Michael J. Blackman, Cally Roper, Christopher J. Drakeley, Arnab Pain, Colin J. Sutherland, Martin L. Hibberd, Susana Campino, Taane G. Clark

**Affiliations:** 1 Faculty of Infectious and Tropical Diseases, London School of Hygiene and Tropical Medicine, London, United Kingdom; 2 Genome Institute of Singapore, Biopolis, Singapore; 3 The Francis Crick Institute, London, United Kingdom; 4 King Abdullah University of Science and Technology, Thuwal, Kingdom of Saudi Arabia; 5 Faculty of Epidemiology and Population Health, London School of Hygiene and Tropical Medicine, London, United Kingdom; Ospedale San Pietro Fatebenefratelli, ITALY

## Abstract

The macaque parasite *Plasmodium knowlesi* is a significant concern in Malaysia where cases of human infection are increasing. Parasites infecting humans originate from genetically distinct subpopulations associated with the long-tailed (*Macaca fascicularis (Mf))* or pig-tailed macaques (*Macaca nemestrina (Mn))*. We used a new high-quality reference genome to re-evaluate previously described subpopulations among human and macaque isolates from Malaysian-Borneo and Peninsular-Malaysia. Nuclear genomes were dimorphic, as expected, but new evidence of chromosomal-segment exchanges between subpopulations was found. A large segment on chromosome 8 originating from the *Mn* subpopulation and containing genes encoding proteins expressed in mosquito-borne parasite stages, was found in *Mf* genotypes. By contrast, non-recombining organelle genomes partitioned into 3 deeply branched lineages, unlinked with nuclear genomic dimorphism. Subpopulations which diverged in isolation have re-connected, possibly due to deforestation and disruption of wild macaque habitats. The resulting genomic mosaics reveal traits selected by host-vector-parasite interactions in a setting of ecological transition.

## Author summary

*Plasmodium knowlesi*, a common malaria parasite of long-tailed and pig-tailed macaques, is now recognized as a significant cause of human malaria, accounting for up to 70% of malaria cases in certain areas in Southeast Asia including Malaysian Borneo. Rapid human population growth, deforestation and encroachment on wild macaque habitats potentially increase contact with humans and drive up the prevalence of human *Plasmodium knowlesi* infections. Appropriate molecular tools and sampling are needed to assist surveillance by malaria control programmes, and to understand the genetics underpinning *Plasmodium knowlesi* transmission and switching of hosts from macaques to humans. We report a comprehensive analysis of the largest assembled set of *Plasmodium knowlesi* genome sequences from Malaysia. It reveals genetic regions that have been recently exchanged between long-tailed and pig-tailed macaques, which contain genes with signals indicative of rapid contemporary ecological change, including deforestation. Additional analyses partition *Plasmodium knowlesi* infections in Borneo into 3 deeply branched lineages of ancient origin, which founded the two divergent populations associated with long-tailed and pig-tailed macaques and a third, highly diverse population, on the Peninsular mainland. Overall, the complex *Plasmodium* parasite evolution observed and likelihood of further host transitions are potential challenges to malaria control in Malaysia.

## Introduction

*Plasmodium knowlesi*, a common malaria parasite of long-tailed *Macaca fascicularis (Mf)* and pig-tailed *M*. *nemestrina (Mn)* macaques in Southeast Asia, is now recognized as a significant cause of human malaria. A cluster of human *P*. *knowlesi* cases were reported from Malaysian Borneo in 2004 **[1],** but now human infections are known to be widespread in Southeast Asia **[2,3],** and have been reported in travellers from outside the region **[2,4]**. Clinical symptoms range from asymptomatic carriage to high parasitaemia with severe complications including death **[5,6].** As rapid human population growth, deforestation and encroachment on remaining wild macaque habitats potentially increases contact with humans **[7]**, in Southeast Asian countries *P*. *knowlesi* is now coming to the attention of national malaria control and elimination programmes that have hitherto focused on *P*. *vivax* and *P*. *falciparum* **[2]**.

*P*. *knowlesi* commonly displays multi-clonality in humans and macaques, and analysis of microsatellite markers, *csp*, *18S rRNA*, and *mtDNA* sequences indicates no systematic differences between human and macaque isolates from Malaysian Borneo **[8]**. Whole genome-level genetic diversity among *P*. *knowlesi* from human infections in Sarikei in Sarawak demonstrates substantial dimorphism extending over at least 50% of the genome **[9].** This finding is supported by analysis of microsatellite diversity in parasites from *Mf*, *Mn* and human infections across Peninsular and Borneo Malaysia **[10]**. It also provides evidence that the two distinct genome dimorphs reflect adaptation to either of the two host macaque species, although no evidence of a complete barrier in primate host susceptibility was found **[10].** A third genome cluster has been described from geographically distinct Peninsular Malaysia **[11, 12, 13, 14].**

Studies of *mtDNA* have revealed that ancestral *P*. *knowlesi* predates the settlement of *Homo sapiens* in Southeast Asia, the evolutionary emergence of *P*. *falciparum* and *P*. *vivax*, and underwent population expansion 30–40 thousand years ago **[8].** Diversity at the genomic level is thus likely to reflect host- and geography-related partitioning during this expansion, as well as additional recent complexity due to contemporary changes in host and vector distributions during ongoing ecological transition in the region **[15].** Several *Anopheles* species, all from the Leuchosphyrus group, are capable of transmitting *P*. *knowlesi* malaria, including *A*. *latens* and *A*. *balbacensis* in Malaysian Borneo **[16, 17, 18],** *A*. *hackeri* and *A*. *cracens* in Peninsular Malaysia **[19]** and *A*. *dirus* in southern Vietnam **[20].** It is thus likely that patterns of genome diversity in natural populations of *P*. *knowlesi* reflect partitioning among both Dipteran and primate hosts occurring on varying time-scales through the evolutionary history of the species. Such partitioning can plausibly prevent or reduce panmictic genetic exchange.

Genomic studies of *P*. *knowlesi* to date have considered nuclear gene diversity and dimorphism among naturally-infected human hosts, and macaque-derived laboratory-maintained isolates from the 1960s **[10, 12]**. However, these studies did not consider non-nuclear organellar genomes in the mitochondrion and apicoplast of malaria parasites, which are non-recombinant and uniparentally inherited, and can provide evidence of genome evolution on a longer timescale **[21].** Recombination barriers among insect and primate hosts may have less impact on sequence diversity in the organellar genomes of *P*. *knowlesi*. Utilising a new *P*. *knowlesi* reference genome generated using long-read technology **[22]** we performed a new analysis of all available nuclear and non-nuclear genome sequences. Patterns of polymorphisms were analysed to identify evolutionary signals of both recent and ancient events associated with the partitioning of the di- or tri-morphic genomes previously reported.

## Results

### Sequence data reveals multiplicity of infection

Raw short-read sequence data from all available *P*. *knowlesi* isolates **(S1 Fig)** were mapped to a new reference genome **[22]** from the human-adapted *P*. *knowlesi* line A1-H.1 genome **[23]**, yielding an average coverage of ~120-fold across 99% of the reference genome (**S1 Table**), and 1,632,024 high quality SNPs. The high density of point mutations (1 every 15bp) in *P*. *knowlesi* compared to *P*. *vivax* and *P*. *falciparum* has been previously noted **[10].** Seven macaque-derived isolates were found to have high multiplicity of infection (**S2 Fig**), and were excluded, leaving an analysis set of 60 isolates.

### Population structure analysis reveals new natural genetic exchange

**Fig 1:**
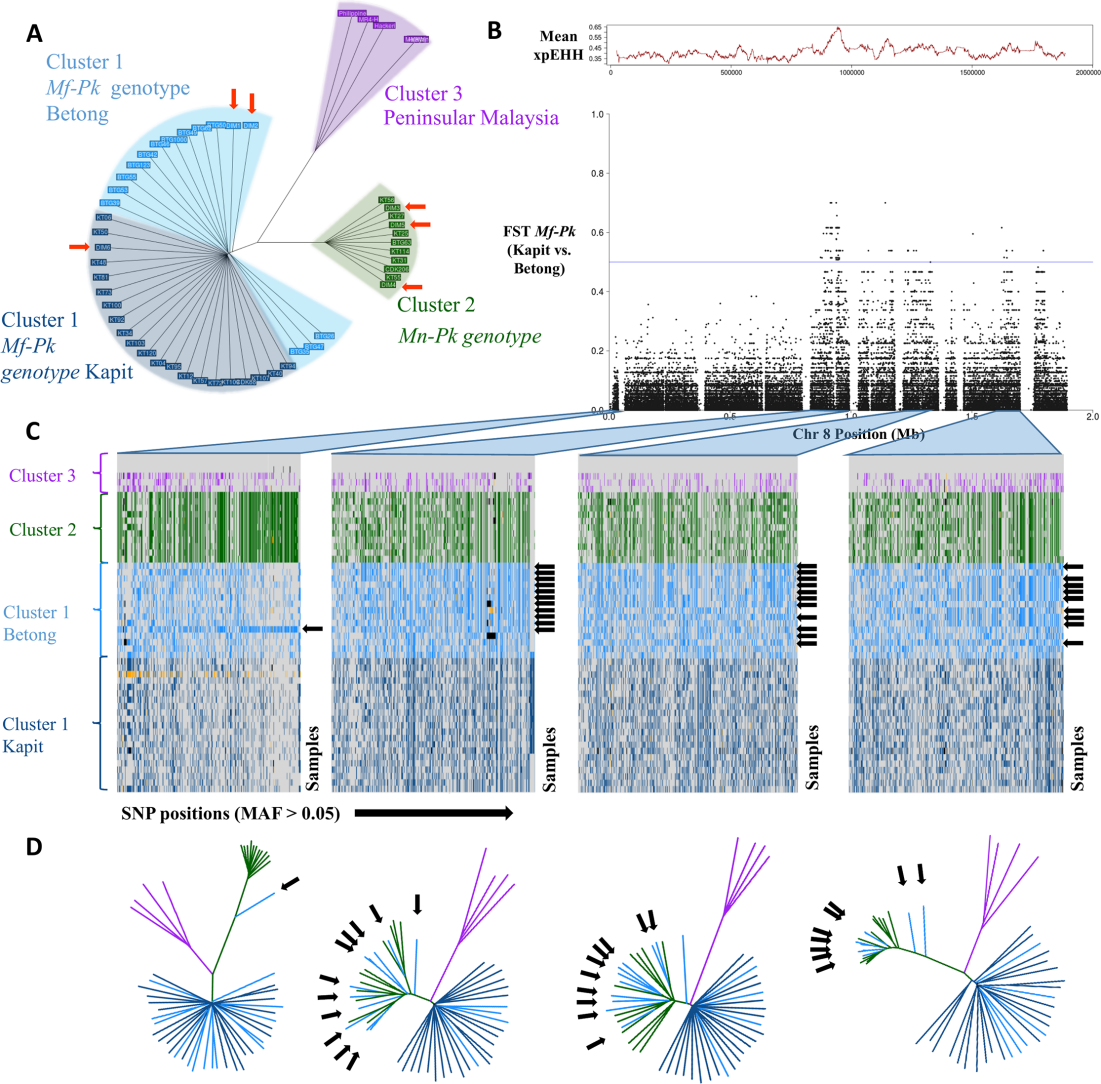
Whole genome population structure and evidence of genetic exchange in chromosome 8. **A)** Neighbour joining tree constructed using 1,632,024 genome-wide SNPs across the 60 *P*. *knowlesi (Pk)* samples. The tree shows two levels of resolution involved in the clustering of genotypes. The first level differentiates Peninsular Malaysia samples (Cluster 3, purple) from the Malaysian-Borneo host-related *Pk* genotypes (Cluster 1, *M*. *fascicularis* macaques (*Mf-Pk*), blue; Cluster 2, *M*. *nemestrina* macaques (*Mn-Pk*), green). The second level differentiates within Cluster 1, where *Mf-Pk* genotypes fall in subgroups from Betong (light blue) and from Kapit (dark blue). Samples from Sarikei have been highlighted using orange arrows. **B)** Allele frequency differences between Betong and Kapit regional subgroups of the *Mf-Pk* genotype in chromosome 8 SNPs using the population differentiation measure *F*_*ST*._ There is high differentiation (*F*_*ST*_ > 0.4) in several regions across chromosome 8 (0.85-1Mb, 1.2Mb-1.35Mb, and 1.6–1.7Mb), and these signals overlap with strong evidence of recent positive selection, measured by the average XP-EHH calculated in 1kbp windows (red trace above). **C)** Haplotype plots for all samples (y-axis) at common SNP positions (MAF >5%, x-axis) highlighting the regions with abnormally high *F*_*ST*_ values (0.85-1Mb, 1.2Mb-1.35Mb, and 1.6–1.7Mb), as well as the low *Fst* region spanning from 0.1 to 0.2Mb for comparison. The black arrows indicate samples with the *Mf-Pk* genotype from Betong present with a *Mn-Pk* Cluster 2-like haplotype. These patterns are indicative of genetic exchange between the *Mf-Pk* and *Mn-Pk* genotype clusters, which is supported by the neighbour joining trees included in **D)**. Missing calls are coloured in black and mixed calls are coloured in yellow. **D)** Neighbour joining trees constructed using SNPs in each of the regions in **C**). The trees show clear clustering of *Mf-Pk* Betong samples with the *Mn-Pk* genotype cluster in the genetic regions of abnormal *F*_*ST*_ (2^nd^, 3^rd^ and 4^th^ trees) compared to the 1^st^ tree where only sample DIM2 presents introgression.

SNP-based neighbour-joining tree analysis revealed three subpopulation groups that coincide with isolates presenting the *Mf*-associated *P*. *knowlesi* genotype (*Mf*-*Pk*, Borneo Malaysia, Cluster 1), the *Mn*-associated *P*. *knowlesi* genotype (*Mn*-*Pk*, Borneo Malaysia, Cluster 2) **[10, 11, 12, 14]**, and older Peninsular Malaysia strains (Cluster 3) (**Fig 1A**). Within Cluster 1 we observed two geographic sub-groups that coincide with Kapit and Betong regions in Malaysian Borneo. The samples from Sarikei region (DIM prefix), geographically located equidistant between Kapit and Betong, fall into either cluster (**S3 Fig**). Overall, the regional clusters from Kapit and Betong were more genetically similar to each other (mean fixation index *F*_*ST*_ 0.03, **S4 Fig**) than were the host-associated clusters (Cluster 1 vs. 2, mean *F*_*ST*_ 0.21). However, a significant chromosomal anomaly was identified that differentiated the Kapit and Betong *Mf-Pk* subgroups; this occurred in a multi-gene region on chromosome 8 (~500 SNPs with *F*_*ST*_ values >0.4; **Fig 1B; S4 Fig**).

### Signatures of introgression events in chromosome 8

To explore the anomaly in chromosome 8, individual haplotypes and neighbour-joining trees were constructed across several loci (**Fig 1C and Fig 1D**) revealing two very distinct patterns. The first pattern was observed in the chromosomal sections with low genetic diversity between the two *Mf-Pk* regional clusters (*F*_*ST*_ < 0.2, **Fig 1B)**. The tree structure for these genomic regions (**Fig 1D,** 1st tree) mimics that of the genome-wide tree in **Fig 1A.** Strong haplotype differentiation between the host-associated Clusters 1 (*Mf-Pk)* and 2 *(Mn-Pk)* was confirmed in the SNP-based profiles (**Fig 1C,** 1st column).

A second pattern was observed in regions of chromosome 8 with distinct genetic differentiation between Kapit and Betong subgroups (*F*_*ST*_ > 0.4). Many *Mf-Pk* Betong subgroup isolates presented segments almost identical to chromosome 8 sequences of the *Mn-Pk* genotype from Cluster 2 (**Fig 1D,** 2^nd^, 3^rd^ and 4^th^ trees). This exchange is supported by the SNP-based haplotype patterns, where a distinct haplotype in the Betong samples is Cluster 2-like (**Fig 1C**, 2^nd^, 3^rd^ and 4^th^ columns, black arrows), suggesting the introgression of large chromosomal regions (up to 200Kb) between *Mf-Pk* (Cluster 1) and *Mn-Pk* (Cluster 2). This is consistent with a very recent event of natural genetic exchange between these subgroups of *P*. *knowlesi* recently isolated from human infections. The high frequency of the new haplotype (73%) in the Betong subgroup suggests that it is under (recent) strong selection pressure in this region. The presence of differences in extended haplotype homozygosity between the recombinant and non-recombinant regional *Mf-Pk* subpopulations provides additional evidence of recent positive selection (XP-EHH peak, P<0.0001) in a region of increased population differentiation (*F*_*ST*_ > 0.4, **Fig 1B**).

The functional nature of genes in chromosome 8 involved in these putative introgression events was investigated (*F*_*ST*_ > 0.4, **Table 1**), and found to include loci that are important in the vector component of the *Plasmodium* life cycle. For example, *cap380* (*PKNH_0820800*, 101 SNPs with *F*_*ST*_ > 0.4) encodes a protein expressed in the external capsule of the oocyst. This gene is essential in the maturation from ookinete into oocyst in *P*. *berghei*, and is assumed to assist in evasion of mosquito immune mechanisms **[24].** Another gene, *PKNH_0826900* (19 SNPs) encodes for the circumsporozoite- and TRAP-related protein (CTRP), which has an established role in ookinete motility *in P*. *berghei* and is essential for binding to and invading the mosquito midgut **[25].** Further, homologues of *PKNH_0826400* (21 SNPs) display increased transcription levels in ookinete and gametocyte V sexual stages in both *P*. *falciparum* **[26]** and *P*. *berghei* **[27]** compared to the asexual ring stage (fold change of at least 2). The transcriptomic profiles of these strongly selected genes are shown in **S5 Fig**.

**Table 1:** Genes located within the chromosome 8 regions of genetic exchange and transcriptional changes.

No. SNPs*	Gene name *(PKNH_)*	Product	*P*.*falcip* Ortholog *(PF3D7_)*	*P*. *berghei* *ortholog (PBANKA_)*	*P*.*falcip* (Ring vs. Ookinete)*	*P*.*berghei* (Ring vs. Ookinete)**
218	Non-genic	-	-	-	-	-
**101**	***0820800***	**oocyst capsule protein (Cap380)**	***0320400***	***1218100***	**Yes**	**Yes**
**21**	***0826400***	**conserved Plasmodium membrane protein**	***0315700***	***0413500***	**Yes**	**Yes**
**19**	***0826900***	**circumsporozoite- & TRAP-related (CTRP)**	***0315200***	***0412900***	**Yes**	**Yes**
15	*0819600*	N-acetylglucosaminephosphotransferase	*0321200*	*1217300*	**Yes**	**No**
11	*0820300*	nicotinamidase	*0320500*	*1218000*	**Yes**	**No**
9	*0828500*	conserved Plasmodium protein	*0313700*	*0411400*	**Yes**	**No**
7	*0819500*	conserved Plasmodium protein	*0321300*	*1217200*	**No**	**No**
7	*0820200*	conserved Plasmodium protein	*0320600*	*1217900*	**Yes**	**No**
7	*0828400*	conserved Plasmodium protein	*0313800*	*0411500*	**Yes**	**No**
7	*0837200*	conserved Plasmodium protein	*0305500*	*0404000*	**No**	**No**
6	*0822900*	conserved Plasmodium protein	*0318500*	*0806700*	**Yes**	**No**
5	*0836500*	activator of Hsp90 ATPase (AHA1)	*0306200*	*0404600*	**Yes**	**No**
5	*0839000*	inner membrane complex protein 1e	*0304100*	*0402700*	**Yes**	**No**
4	*0819700*	conserved Plasmodium protein	*0321100*	*1217400*	**Yes**	**No**
4	*0820100*	signal peptidase complex subunit 2 (SPC2)	*0320700*	*1217800*	**Yes**	**No**
4	*0823100*	conserved Plasmodium protein	*0318300*	*0806900*	**No**	**No**
4	*0836000*	membrane magnesium transporter	*0306700*	*0405100*	**No**	**No**
4	*0839100*	inner membrane complex protein 1a	*0304000*	*0402600*	**No**	**Yes**
3	*0820900*	T-complex protein 1 subunit epsilon (CCT5)	*0320300*	*1218200*	**No**	**No**
3	*0828300*	conserved Plasmodium protein,	*0313900*	*0411600*	**Yes**	**No**
3	*0838400*	conserved Plasmodium protein,	*-*	*-*	**No**	**No**
2	*0821000*	CPW-WPC family protein	*0320200*	*1218300*	**Yes**	**No**
2	*0821500*	ABC transporter I family member 1 (ABCI3)	*0319700*	*1218800*	**No**	**No**
2	*0822800*	cleavage and polyadenylation factor	*0318600*	*0806600*	**No**	**No**
2	*0824500*	conserved Plasmodium protein	*0317300*	*0807900*	**No**	**No**
2	*0825900*	conserved Plasmodium protein	*0316200*	*0414000*	**Yes**	**Yes**
2	*0827400*	zinc finger protein	*0314700*	*0412400*	**Yes**	**Yes**
2	*0828700*	conserved Plasmodium protein	*0313500*	*0411200*	**Yes**	**No**
2	*0837600*	conserved Plasmodium protein	*0305100*	*0403600*	**Yes**	**No**
2	*0838500*	circumsporozoite (CS) protein (CSP)	*0304600*	*0403200*	**No**	**Yes**
2	*0838800*	conserved Plasmodium protein	*0304300*	*0402900*	**No**	**Yes**
2	*0839200*	phosphatidylethanolamine-binding protein	*0303900*	*0402500*	**Yes**	**No**
1	*0819100*	conserved Plasmodium protein	*0321700*	*1216800*	**Yes**	**No**
1	*0819200*	ATP-dependent RNA helicase	*0321600*	*1216900*	**Yes**	**No**
1	*0819400*	protein kinase, putative	*0321400*	*1217100*	**Yes**	**No**
1	*0819900*	histone H2A variant, putative (H2A.Z)	*0320900*	*1217600*	**No**	**No**
1	*0820000*	ATP-dependent RNA helicase DDX6 (DOZI)	*0320800*	*1217700*	**Yes**	**No**
1	*0821200*	protein phosphatase inhibitor 2	*0320000*	*1218500*	**Yes**	**No**
1	*0821400*	conserved Plasmodium protein	*0319800*	*1218700*	**No**	**Yes**
1	*0821600*	elongation factor 1	*-*	*1218900*	**No**	**No**
1	*0821700*	RNA-binding protein	*0319500*	*0805800*	**No**	**No**
1	*0823000*	conserved Plasmodium protein	*0318400*	*0806800*	**Yes**	**Yes**
1	*0823500*	DNA-directed RNA polymerase II subunit	*1329000*	*0807000*	**No**	**No**
1	*0824700*	6-cysteine protein	*0317100*	*0808100*	**Yes**	**Yes**
1	*0824900*	E3 ubiquitin-protein ligase, putative	*0316900*	*0808300*	**No**	**No**
1	*0825200*	formate-nitrite transporter, putative (FNT)	*0316600*	*0414400*	**No**	**No**
1	*0826700*	conserved Plasmodium protein	*0315400*	*0413200*	**Yes**	**No**
1	*0827000*	eukaryotic translation initiation factor 4E	*0315100*	*0412800*	**No**	**No**
1	*0827300*	conserved Plasmodium protein,	*0314800*	*0412500*	**No**	**Yes**
1	*0836300*	FAD-dependent glycerol-3-phosphate	*0306400*	*0404800*	**Yes**	**No**
1	*0836600*	conserved Plasmodium protein	*0306100*	*0404500*	**No**	**No**
1	*0838900*	EH (Eps15 homology) protein	*0304200*	*0402800*	**Yes**	**No**
1	*0839300*	IBR domain protein	*0303800*	*0402400*	**No**	**No**

* Cells in green (with “Yes”) imply that the *P*. *falciparum* orthologue (Column 4) of the *P*. *knowlesi* gene (Column 2) has at least a two-fold change difference in the transcriptional signals from *P*. *falciparum* when comparing Ring vs. Ookinete stages

** similar to *, but refers to the *P*. *berghei* orthologue (Column 5) having at least a two-fold change difference in the transcriptional signals; **bolded genes** have >10 SNPs with F_ST_ > 0.4.

### Genome-wide evidence of genetic exchange events in *P*. *knowlesi*

By applying a combination of neighbour joining trees and SNP diversity analysis across 50 Kbp windows, we identified that 33/60 isolates show clear evidence of genetic exchange between Clusters 1 and 2 (**S2 Table**). Regions involved in exchange (recombination) (137/494 regions, 86% contained an ookinete related gene) showed evidence of enrichment for ookinete-expressed genes compared to other (non-recombinant) chromosome regions (357/494 regions, 77% contained an ookinete related gene) (Chi Square P = 0.03). One such region in chromosome 12 included the *Pf47-like (PKH_120710)* gene, where the orthologue in *P*. *falciparum* is a known mediator of the evasion of the mosquito immune system **[28]**. Furthermore, it has been shown that a change in haplotype in this gene in a *P*. *falciparum* isolate is sufficient to make it compatible to a different mosquito species **[28]**. Nearly half (45%) of isolates from Betong presented with a recombinant profile in *PKH_120710*.

In general, the genetic exchanges generated differing levels of mosaicism in each population and among individual isolates across all chromosomes (**S6 Fig**). One isolate from Sarikei with the *Mf* genome dimorph type (DIM2) appeared to harbour *Mn-*type introgressed sequences in 8% of the genome, occurring across 6 chromosomes (6, 7, 8, 9, 11 and 12), including an almost complete *Mn-*type chromosome 8. Of the 33 samples with evidence of exchanges, 13 were from the Betong region, 14 from Kapit and 6 from Sarikei, which indicates that the events are not geographically restricted. Although, the majority of genetic exchange events involve the integration of *Mn-*type motifs into *Mf-*type genomes, introgression in the opposite direction was also observed, but on a smaller scale and at lower frequency.

### Organellar genomes also reflect genetic exchange events

**Fig 2:**
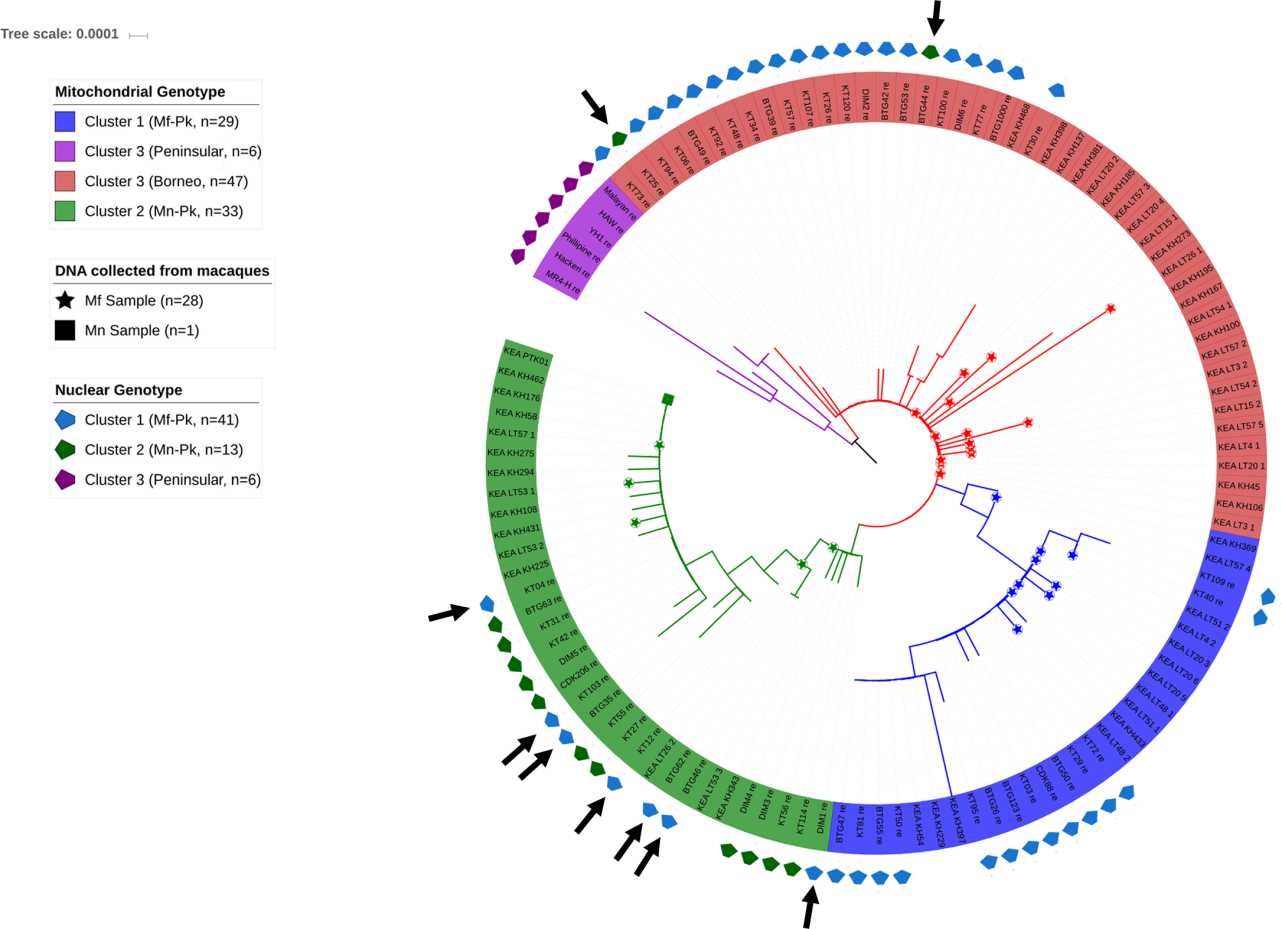
Phylogenetic tree constructed from *P*. *knowlesi* mitochondrial sequences for the 60 whole genome sequenced samples and 54 published others [6] sourced from human, *M*. *nemestrina (Mn)* and *M*. *fascicularis (Mf)* samples. The mitochondrial genotype groups defined here are cross-referenced to the nuclear genotypes in **Fig 1A** (pentagons in the outer ring, missing pentagons relate to the 54 samples with only mitochondrial sequence data **[6]**). Samples sourced from the different macaques are highlighted in the tree branches. The tree shows three main subpopulations: (i) two clades including Peninsular Malaysia (Peninsular nuclear genotype, Cluster 3, purple) and Borneo Malaysia (mix of *Mf-Pk* and *Mn-Pk* nuclear genotypes, Cluster 1 and 2, red) presenting a very similar mitochondrial haplotype; (ii) the majority of the samples with a *Mn-Pk* nuclear genotype together with the only sequence obtained from a *Mn* sample (Cluster 2, green); (iii) samples with a *Mf-Pk* nuclear genotype (Cluster 1, blue). These clusters are consistent with microsatellite-based trees **[12].** The presence of monkey samples spread throughout the tree indicates that none of the mitochondrial genotypes groups are human-specific, consistent with microsatellite-based analysis **[9]**. Black arrows indicate the presence of samples with mismatched nuclear and mitochondrial subtypes.

The mitochondrial and apicoplast genomes of each *P*. *knowlesi* isolate was interrogated for signals of evolutionary history over longer time-scales, as in previous studies **[21, 29, 30].** Combining the mitochondrial sequence data from the 60 *P*. *knowlesi* isolates from this study together with 54 previously published mitochondrial sequences including human and both *Mn* and *Mf* samples **[9]**, we generated a phylogenetic tree (**Fig 2).** This tree shows four clades (shown in purple, red, blue and green). To interpret these clades, they were cross-referenced to the previously defined 3 nuclear genotypes (Clusters 1 to 3) and the host contributing the sample (human, macaque-type). The red and purple clades possess similar mitochondrial haplotypes as highlighted by their inter-cluster average *F_ST_* (red vs. purple: average *F_ST_* = 0.16), which is lower than comparisons including the other two clusters (red or purple vs. blue or green: average *F_ST_* > 0.18). The purple clade consists of cultured isolates from Peninsular Malaysia, and is associated with the Peninsular nuclear genotype (Cluster 3). The red and green clades each contain a mixture of Borneo Malaysia samples from both humans and macaques with nuclear genotypes from Clusters 1 and 2. The green clade also includes the only sequence sourced from a *M*. *nemestrina* host. The blue clade contains samples from humans and macaques, all with Cluster 1 nuclear genotypes. The divergence of these mitochondrial clades from their common ancestor was estimated to be 72k years ago, and younger than the previous the estimate of 257k but within error **[8]**. Furthermore, the presence of monkey-derived sequences spread across the tree seems to indicate that none of the mitochondrial genotypic groups found is human-specific as all have also been observed in macaques, also consistent with previous findings **[9].**

Using the common SNPs (280/425 with MAF > 5%: apicoplast 252, mitochondria 28 SNPs) in the 60 isolates with the sequence data we confirmed that the organellar genomes are co-inherited (mean pairwise organellar linkage disequilibrium D’ = 0.99). SNP-based haplotype profile analysis (**S7A Fig)** revealed clustering that is consistent with the three main clusters seen in **Fig 2**. Similarly, a phylogenetic tree constructed using only apicoplast SNPs (**S7B Fig**) is congruent with the mitochondrial based tree (**Fig 2**). The presence of mismatched nuclear and organellar type genomes in two of the three clusters **(black arrows in Fig 2)** and the presence of such mismatched samples with little or no evidence of nuclear genome recombination suggests ancient genetic exchange events between distinct lineages. The nuclear footprints of such exchanges are likely to have been broken down by recombination over time. We observed a significant incongruence between the robust phylogenetic tree topologies based on organellar and nuclear genome SNPs (Shimodaira-Hasegawa test P = 0.001; Templeton test P = 0.003) (**Fig 2**). These results from organellar and nuclear genomes, in a small but geographically diverse set of *P*. *knowlesi*, indicate that there have been several genetic exchanges between the host-associated clusters in Malaysian Borneo.

## Discussion

*P*. *knowlesi* is now the major cause of malaria in Malaysian Borneo, but the biology of the parasite [**15, 22, 23**], host and vector interactions, and disease distribution and epidemiology [**19, 31, 32]** are not well understood. The availability of a new high-quality reference sequence and a more robust approach to MOI were used to re-evaluate the previously described peninsular and macaque-associated subpopulations of *P*. *knowlesi* parasites. We report two major new findings. First, clear evidence of natural genetic exchanges between the divergent *Mf-* and *Mn*-associated subpopulations of *P*. *knowlesi*, including a major segment of introgression on chromosome 8, is presented. Second, the presence of haplotype sub-divisions in the organellar genomes that do not map onto the subpopulations implied by nuclear genome analysis indicate that exchange events have previously occurred in non-recent history. A similar multi-tiered pattern of evolution among nuclear and organellar genomes has been found in *Trypanosoma cruzi*, an unrelated protozoan parasite with a mammalian host-insect vector life cycle **[29, 30].**

Unexpectedly, observed mosaicism and population differentiation signals were not encountered equally across the *P*. *knowlesi* nuclear genome, but were particularly prominent on chromosome 8, with genes expressed in mosquito stages over-represented. For example, the majority (73%) of *Mf-*associated isolates from Betong harboured the *Mn-*associated allele of the oocyst-expressed c*ap380* gene, which differs at 101 positions from the allele found in the *Mf*-associated cluster. This is essential for ookinete to oocyst maturation and therefore for the transmission of the parasite during the vector stage **[24, 25];** here, we identify signals of recent selective pressure on this locus (**Fig 1B**). Other vector-related genes were identified within the introgressed segment, and point towards strong evolutionary selection pressure on the parasites driven by the transmitting *Anopheles* vector species. Such effects have been found in *P*. *falciparum* **[28]** and *P*. *vivax* genomes **[33],** and highlight the importance of understanding the distribution of the different *Anopheles* vector species, their host feeding preferences, and their interactions with the parasite in highly dynamic and complex environments such as the ecological niche of *P*. *knowlesi*.

Nearly 80% of Malaysian Borneo has undergone deforestation or agricultural expansion, which have driven habitat modification affecting both macaque and *Anopheles* host species, and the proximity of humans to both **[8, 31].** Furthermore, studies have predicted that *Mn* predominantly inhabits forested areas while *Mf* reside in more cosmopolitan areas, which include croplands, vegetation mosaics, rubber plantations and forested areas **[8, 34].** The main genomic exchange event on chromosome 8 involves essential vector-related genes and is pin-pointed geographically to the Betong area. This region has undergone significant forest degradation due to expansion of industrial plantations in the recent years **[35]**. These types of environmental changes have been previously related to alterations in the vector species distribution in Malaysia, leading to malaria epidemics **[36]**. Environmental changes also affect macaque habitats, and increase the opportunities for human-macaque interaction **[31],** but selection events highlighted in this study seem to primarily reflect adaptation of the parasite to changes in mosquito distribution or to recent changes in the vectorial capacity of the existing vectors. The depth, breadth and spread of the genetic exchanges observed in three different areas (Betong, Kapit and Sarikei) in Sarawak highlight the potential importance of these events for parasite adaptation in both vertebrate and invertebrate species.

Although, the level of genetic diversity between *Mf-* and *Mn*-associated *P*. *knowlesi* has some similarity to that observed between *P*. *ovale curtisi* and *P*. *o*. *wallikeri*, now considered separate species **[37],** the evidence of recombination and genetic exchanges observed in this study precludes species designation, as reproductive isolation is not complete. Nevertheless, better understanding of *P*. *knowlesi* population structure could aid future studies across the regions where human populations have been identified at risk of infection including both symptomatic and asymptomatic cases **[4, 38, 39].** This would assist with characterising and tracking subpopulations and genetic exchanges, and provide a flexible framework for better understanding *P*. *knowlesi* diversity across the region.

Our work has provided insight into *Plasmodium* parasite evolution. It has been suggested that malaria parasites have survived using either adaptive radiation where host switching plays a key role **[40],** or alternatively adaptation to complex historical and geographical environments leading to speciation **[41].** *Plasmodium* species in non-human natural conditions in the absence of drug selection pressure have a wide range of possible hosts **[41, 42].** The *P*. *knowlesi* data has shown that geographical or ecological isolation of the different hosts over an extended time can generate subgroups of parasites with substantial genetic differentiation, but capable of recombining when in contact **[12, 30, 31]**. This pattern has a major impact on the parasite genome, as illustrated by the profound chromosome mosaicism observed among our study isolates. Our data suggest that the broad host specificity of some of the *Plasmodium* species are important drivers of parasite genomic diversity. In *P*. *knowlesi* this means that genetic divergence is enabled not only by long-term geographic isolation, as is the case between Peninsular and Bornean isolates, but also via the isolation afforded by extended transmission cycles within different primate hosts. The genetic trimorphism suggests that the separate macaque hosts provides sufficient genetic isolation to allow for host specific adaptations to occur, even within relatively small geographic areas. Furthermore, the possibility of recombination between partially differentiated parasite genomes increases opportunities for new adaptation, including further host transitions, and can only make malaria control more difficult. Genome-level studies on *P*. *knowlesi* isolates from *Mf* and *Mn* across the parasite’s geographic range are now needed to test the generalizability of this remarkable conclusion.

## Materials and methods

### *P*. *knowlesi* sequence data

Raw sequence data were downloaded for 48 isolates from Kapit and Betong in Malaysian Borneo **[11],** 6 isolates from Sairikei in Malaysian Borneo **(S1 Fig) [9]** and 6 long-time isolated lines, maintained in rhesus monkeys sourced originally from Peninsular Malaysia and Philippines **[11]**. The sequence data accession numbers can be found in **S1 Table**. The samples were aligned against the new reference for the human-adapted line A1-H.1 (pathogenseq.lshtm.ac.uk/knowlesi_1, accession number ERZ389239, [**22**]) using *bwa-mem* **[43]** and SNPs were called using the *Samtools* suite **[44],** and filtered for high quality SNPs using previously described methods **[45, 46].** In particular, the SNP calling pipeline generated a total of 2,020,452 SNP positions, which were reduced to 1,632,024 high quality SNPs after removing those in non-unique regions, and in low quality and coverage positions. Samples were individually assessed for detecting multiplicity of infection (MOI) using: (i) *estMOI* **[47]** software, and (ii) quantifying the number of positions with mixed genotypes (if more than one allele at a specific position have been found in at least 20% of the reads **[46]**). The measures led to correlated results (*r*^*2*^ = 0.8), which highlighted the robustness of these two methods. Samples were classified into three subcategories: (i) single infections (> = 98% genome showing no evidence of MOI and < = 1/10,000 SNP positions with mixed genotypes), (ii) low MOI (>85% genome showing no evidence of MOI and < = 4/10,000 SNPs positions with mixed genotypes); (iii) high MOI (<85% genome showing no evidence of MOI, and > 4/10,000 SNPs positions with mixed genotypes). Samples with high MOI were removed from subsequent analyses.

### Population genetics analysis

For comparisons between populations, we first applied the principal component analysis (PCA) and neighbourhood joining tree clustering based on a matrix of pairwise identity by state values calculated from the SNPs. We used the ranked *F*_*ST*_ statistics to identify the informative polymorphism driving the clustering observed in the PCA **[48].** Finally, we created haplotype plots using only SNP positions with MAF > 0.05 over all the populations, and displayed each sample as a row to allow closer inspection of the chromosome regions where interesting recombination events are observed. The XP-EHH metric **[49]** implemented within the *rehh* R package was used to assess evidence of recent relative positive selection between regional clusters from Kapit and Betong. The results were smoothed by calculating means in 1 Kbp windows, where windows overlapped by 250bp. The *raXML* software (v.8.0.3, 1000 bootstrap samples) was used to construct robust phylogenetic trees (90% bootstrap values > 95) for nuclear and organellar SNPs. Estimates of divergence times for subpopulations was based on a Bayesian Markov Chain Monte Carlo (MCMC) (BEAST, v.1.8.1) approach applied to mitochondrial sequences, with identical parameters settings to those described elsewhere **[8].** The Shimodaira-Hasegawa **[50]** and the Templeton **[51]** tests were used to detect incongruence between the tree topologies.

### Identification of introgressed regions in the different chromosomes

In order to identify regions that have undergone introgression we calculated the pairwise SNP diversity (π) of each sample against all the Borneo samples using a 50 Kbp sliding window. This window size was sufficient to include the required number of SNPs for the robust identification of introgression events. The average π in the *M*. *nemestrina* associated (*Mn-Pk*) and *M*. *fascicularis* associated (*Mf-Pk*) clusters was calculated, leading to two diversity values for each sample (*Mf*_*π*_ and *Mn*_*π*_) and thereby a measure of genetic distance to the average of the two clusters. For *Mf* samples, an increase in the *Mf*_*π*_ and a decrease in *Mn*_*π*_ would mean the sample is more similar to the *Mn-Pk* cluster than the average; vice versa for the *Mf* samples. In order to avoid the identification of spurious events, we applied a threshold of a 0.001 increase in the deviation from the original cluster.

### Characterization of genes under strong selection after recombination

For *P*. *knowlesi* genes of interest, orthologues in *P*. *falciparum* and *P*. *berghei* genomes were identified using *PlasmoDB* (plasmodb.org). Gene expression data (including from the RNAseq platform) for these genes across different stages of the life cycle of the parasite were considered **[26, 27]**. In particular, we compared the average of the asexual blood stages and the sexual ookinete stage, highlighting the genes upregulated with a two-fold change (P<0.000001), for *P*. *falciparum* **[26]** and *P*. *berghei* **[27]**.

## Supporting information

S1 Table Study samples. * Multiplicity of infection (MOI) is % of genome presenting multiplicity of infection; **Group established by whole Genome PCA: *Mf M*. *fascicularis*, *Mn M*. *nemestrina*, *Penin*. *Peninsular;* Rh mac Rhesus macaque, *** evidence of genetic exchange (ExΔ)
(DOCX)

S2 Table 50 Kb regions in the *P*. *knowlesi* genome that present genetic exchanges in the full set of samples. (XLSX)

S1 Fig Geographical source of the *P*. *knowlesi* isolates: Betong (n = 14), Kapit (n = 33) and Sarikei (n = 6). (TIFF)

S2 Fig Evaluation of multiplicity of infection (MOI) using mixed genotype calls (x-axis) and the estMOI read-pair haplotype counting approach [45] (y-axis) reveals seven highly non-clonal samples. (TIFF)

S3 Fig Principal components analysis of the *M*. *fascicularis P*. *knowlesi* genotype group (*Mf-Pk*, Cluster 1) confirms that the subgroups from Kapit and Betong are separated. The *Mf-Pk* Sarikei samples (DIM code in orange) cluster with either one of the two groups, which is consistent with the geographic location of Sarikei as an equidistant region between Kapit and Betong. There is increased diversity of Betong samples compared to the Kapit samples.
(TIFF)

S4 Fig Genome-wide differences in allele frequencies (measured using the fixation index (*F*_*ST*_)) between *M*. *fascicularis P*. *knowlesi* genotype groups (*Mf-Pk)* from Kapit and Betong. The comparison shows clear abnormalities in several genomic regions in chromosome 8 shown to be a result of genetic exchange with the *Mn-Pk* genotype.
(TIFF)

S5 Fig Transcriptomic profiles for the orthologues of the introgressed genes under selection pressure. The transcriptomic profiles of the orthologues in *P*. *falciparum* **[26]** and *P*. *berghei* **[27]** for the three genes found to be under strong selection pressure were extracted from PlasmoDB (http://plasmodb.org/plasmo/), including the percentile and the Fragments Per Kilobase of transcript per Million mapped reads (FPKM) plots. These included data for 5 *P*. *berghei* stages (4-hour Ring, 16-hour Trophozoite, 22-hour Schizont, Gametocyte and Ookinete) and 7 *P*. *falciparum* stages (Ring, early Trophozoite, late Trophozoite, Schizont, Gametocyte stage II, Gametocyte stage V and Ookinete), and showing a clear increased expression in mosquito related stages, particularly the ookinete stage.
(TIFF)

S6 Fig Genome distribution of introgression events for each chromosome estimated using SNP diversity in 50Kb sliding windows. **(Left panel)** location of introgressions from *M*. *nemestrina P*. *knowlesi* (*Mn-Pk*) genotype into *M*. *fasciscularis P*. *knowlesi* (*Mf-Pk*) genotypes, a dashed shaded region has been added where at least 1 gene related with the ookinete life stage of the parasite has been identified based on gene expression for the orthologue genes in *P*. *berghei* and/or *P*. *falciparum*. **(Right panel)** location of introgressions from *Mf-Pk* genotype into *Mn-Pk* genotypes.
(TIFF)

S7 Fig **Analysis of organellar mitochondria (MIT) and apicoplast (Api) SNPs confirms clustering into three core haplotype groups a) Haplotype plot for the 36 samples with sufficient coverage across the organellar genomes**. Three clearly defined clusters are present. The first cluster represents the mitochondrial genotype found in the Peninsular strains (purple, n = 5) and a set of 10 samples with a highly related haplotype with the smallest inter-cluster average F_ST_ (average F_ST_ = 0.16) from Borneo Malaysia (represented in red in **Fig 2).** The second cluster (green in **Fig 2**) includes the majority of *M*. *nemestrina P*. *knowlesi (Mn-Pk)* nuclear genotype isolates. The third cluster (blue in **Fig 2**) consists only of isolates with *Mf-Pk* nuclear genotypes. The presence of samples in the other two clusters with mismatched nuclear and organellar genomes indicates that these two subpopulations have undergone genetic exchange. **b) Phylogenetic tree generated using 362 apicoplast SNPs.** The tree shows a very similar pattern of clustering to **Fig 2.**
(TIFF)
