# Supplementary material for: Analysis of nuclear and organellar genomes of Plasmodium knowlesi in humans reveals ancient population structure and recent recombination among host-specific subpopulations
Source: PLoS Genet. 2017 Sep 18;13(9):e1007008. doi: 10.1371/journal.pgen.1007008 (PMC5619863; doi:10.1371/journal.pgen.1007008)
Supplement: S1 Table — * Multiplicity of infection (MOI) is % of genome presenting multiplicity of infection; **Group established by whole Genome PCA: Mf M. fascicularis, Mn M. nemestrina, Penin. Peninsular; Rh mac Rhesus macaque, *** evidence of genetic exchange (ExΔ) (DOCX) [file pgen.1007008.s001.docx]

**S1 Table**

**Study samples**

| **Sample** | **Code** | **Area** | **Host** | **MOI*** | **Group**** | **Total**  **Reads** | **Mapped**  **Reads %** | **Genome**  **covered** | **Cover.**  **Mean** | | **Ex∆***** | **Year** |  |
| --- | --- | --- | --- | --- | --- | --- | --- | --- | --- | --- | --- | --- | --- |
| ERR274221 | DIM1 | Sarikei | Human | 0.8 | *Mf* | 46353062 | 96.9 | 0.99 | 174.1 | Yes | | 2012/3 | |
| ERR274222 | DIM2 | Sarikei | Human | 0.7 | *Mf* | 60189967 | 97.7 | 0.99 | 228.7 | Yes | | 2012/3 | |
| ERR274224 | DIM3 | Sarikei | Human | 0.6 | *Mn* | 42350437 | 97.0 | 0.98 | 161.5 | Yes | | 2012/3 | |
| ERR274225 | DIM4 | Sarikei | Human | 0.5 | *Mn* | 52883838 | 97.2 | 0.98 | 201.6 | Yes | | 2012/3 | |
| ERR366425 | DIM5 | Sarikei | Human | 0.3 | *Mn* | 6391836 | 97.1 | 0.95 | 36.0 | Yes | | 2012/3 | |
| ERR366426 | DIM6 | Sarikei | Human | 0.2 | *Mf* | 6270503 | 94.8 | 0.95 | 34.1 | Yes | | 2012/3 | |
| ERR985372 | BTG1000 | Betong | Human | 0.8 | *Mf* | 30620879 | 66.0 | 0.98 | 79.7 | Yes | | 2012/3 | |
| ERR985374 | BTG26 | Betong | Human | 1.6 | *Mf* | 55120863 | 96.4 | 0.99 | 207.5 | Yes | | 2012/3 | |
| ERR985375 | BTG35 | Betong | Human | 6.8 | *Mf* | 66747737 | 22.7 | 0.98 | 55.4 | Yes | | 2012/3 | |
| ERR985376 | BTG39 | Betong | Human | 9.9 | *Mf* | 50924729 | 79.3 | 0.99 | 157.7 | Yes | | 2012/3 | |
| ERR985377 | BTG42 | Betong | Human | 1.0 | *Mf* | 71277238 | 95.3 | 0.99 | 248.7 | Yes | | 2012/3 | |
| ERR985378 | BTG46 | Betong | Human | 1.4 | *Mf* | 47365570 | 95.6 | 0.99 | 177.7 | Yes | | 2012/3 | |
| ERR985379 | BTG47 | Betong | Human | 1.2 | *Mf* | 40897898 | 95.5 | 0.99 | 153.6 | Yes | | 2012/3 | |
| ERR985380 | BTG49 | Betong | Human | 5.9 | *Mf* | 61989913 | 97.2 | 1 | 234.8 | Yes | | 2012/3 | |
| ERR985381 | BTG50 | Betong | Human | 1.4 | *Mf* | 51234398 | 94.9 | 0.99 | 189.9 | Yes | | 2012/3 | |
| ERR985382 | BTG53 | Betong | Human | 1.3 | *Mf* | 48838502 | 49.6 | 0.98 | 89.3 | Yes | | 2012/3 | |
| ERR985383 | BTG55 | Betong | Human | 3.6 | *Mf* | 40307405 | 85.8 | 0.99 | 126.8 | Yes | | 2012/3 | |
| ERR985384 | BTG62 | Betong | Human | 3.0 | *Mf* | 52753853 | 88.0 | 0.99 | 167.0 | Yes | | 2012/3 | |
| ERR985385 | CDK88 | Kapit | Human | 1.1 | *Mf* | 93728041 | 60.6 | 1 | 213.1 | No | | 2012/3 | |
| ERR985386 | KT03 | Kapit | Human | 22.8 | *Mf* | 52908664 | 22.3 | 0.98 | 45.3 | No | | 2012/3 | |
| ERR985387 | KT04 | Kapit | Human | 2.7 | *Mf* | 48092075 | 91.8 | 0.99 | 173.0 | No | | 2012/3 | |
| ERR985388 | KT06 | Kapit | Human | 1.0 | *Mf* | 68600487 | 92.2 | 0.99 | 247.4 | No | | 2012/3 | |
| ERR985394 | KT12 | Kapit | Human | 1.0 | *Mf* | 26219163 | 95.2 | 0.98 | 99.0 | No | | 2012/3 | |
| ERR985395 | KT26 | Kapit | Human | 50.4 | *Mf* | 45997535 | 86.1 | 1 | 154.6 | No | | 2012/3 | |
| ERR985396 | KT29 | Kapit | Human | 21.0 | *Mf* | 31849384 | 60.9 | 0.99 | 76.4 | No | | 2012/3 | |
| ERR985397 | KT30 | Kapit | Human | 22.3 | *Mf* | 40463921 | 92.0 | 1 | 146.3 | No | | 2012/3 | |
| ERR985398 | KT34 | Kapit | Human | 1.2 | *Mf* | 33387794 | 61.2 | 0.98 | 80.5 | Yes | | 2012/3 | |
| ERR985399 | KT40 | Kapit | Human | 1.3 | *Mf* | 34420982 | 97.0 | 0.99 | 131.2 | No | | 2012/3 | |
| ERR985400 | KT48 | Kapit | Human | 1.0 | *Mf* | 36366112 | 84.6 | 0.99 | 121.4 | No | | 2012/3 | |
| ERR985401 | KT50 | Kapit | Human | 1.0 | *Mf* | 36045889 | 95.3 | 0.99 | 135.9 | Yes | | 2012/3 | |
| ERR985402 | KT57 | Kapit | Human | 0.7 | *Mf* | 56171218 | 92.5 | 0.99 | 202.0 | No | | 2012/3 | |
| ERR985403 | KT72 | Kapit | Human | 0.6 | *Mf* | 52517284 | 95.4 | 0.99 | 193.6 | No | | 2012/3 | |
| ERR985404 | KT73 | Kapit | Human | 1.2 | *Mf* | 52157592 | 94.6 | 1 | 190.2 | No | | 2012/3 | |
| ERR985410 | BTG44 | Betong | Human | 32.7 | *Mn* | 32160549 | 97.1 | 0.99 | 122.9 | No | | 2012/3 | |
| ERR985411 | BTG63 | Betong | Human | 0.8 | *Mn* | 54179835 | 80.4 | 0.98 | 161.6 | Yes | | 2012/3 | |
| ERR985412 | CDK206 | Kapit | Human | 0.8 | *Mn* | 66023442 | 44.2 | 0.99 | 109.4 | Yes | | 2012/3 | |
| ERR985414 | KT25 | Kapit | Human | 0.8 | *Mn* | 65300398 | 93.9 | 0.99 | 242.3 | Yes | | 2012/3 | |
| ERR985415 | KT27 | Kapit | Human | 0.9 | *Mn* | 60931327 | 92.5 | 0.99 | 223.7 | Yes | | 2012/3 | |
| ERR985416 | KT31 | Kapit | Human | 1.2 | *Mn* | 78064720 | 92.6 | 0.99 | 286.4 | Yes | | 2012/3 | |
| ERR985417 | KT42 | Kapit | Human | 29.9 | *Mn* | 41500510 | 95.7 | 0.99 | 157.1 | No | | 2012/3 | |
| ERR985418 | KT55 | Kapit | Human | 0.7 | *Mn* | 54311715 | 89.9 | 0.98 | 191.6 | Yes | | 2012/3 | |
| ERR985419 | KT56 | Kapit | Human | 4.5 | *Mn* | 57630655 | 81.2 | 0.99 | 183.6 | Yes | | 2012/3 | |
| ERR985373 | BTG123 | Betong | Human | 0.7 | *Mf* | 42713162 | 78.6 | 0.98 | 119.3 | Yes | | 2012/3 | |
| ERR985389 | KT100 | Kapit | Human | 2.8 | *Mf* | 41469453 | 86.3 | 0.99 | 126.4 | Yes | | 2012/3 | |
| ERR985390 | KT103 | Kapit | Human | 0.5 | *Mf* | 41673914 | 83.2 | 0.99 | 117.2 | No | | 2012/3 | |
| ERR985391 | KT107 | Kapit | Human | 0.6 | *Mf* | 40562077 | 90.6 | 0.99 | 141.0 | No | | 2012/3 | |
| ERR985392 | KT109 | Kapit | Human | 0.6 | *Mf* | 42267002 | 87.0 | 0.99 | 132.1 | Yes | | 2012/3 | |
| ERR985393 | KT120 | Kapit | Human | 0.5 | *Mf* | 34929904 | 71.1 | 0.98 | 88.5 | No | | 2012/3 | |
| ERR985405 | KT77 | Kapit | Human | 30.9 | *Mf* | 38834680 | 88.7 | 1 | 128.1 | No | | 2012/3 | |
| ERR985406 | KT81 | Kapit | Human | 0.5 | *Mf* | 47157966 | 83.6 | 0.99 | 138.9 | No | | 2012/3 | |
| ERR985407 | KT92 | Kapit | Human | 0.8 | *Mf* | 43617554 | 86.2 | 0.99 | 135.2 | Yes | | 2012/3 | |
| ERR985408 | KT94 | Kapit | Human | 0.6 | *Mf* | 47394270 | 86.8 | 0.99 | 148.1 | Yes | | 2012/3 | |
| ERR985409 | KT95 | Kapit | Human | 3.5 | *Mf* | 50949905 | 80.5 | 0.99 | 134.1 | Yes | | 2012/3 | |
| ERR985413 | KT114 | Kapit | Human | 0.5 | *Mn* | 51040091 | 74.5 | 0.98 | 119.8 | No | | 2012/3 | |
| SRR2221468 | Hackeri | Penin. | Rh mac | 1.2 | *Penin.* | 22542689 | 94.5 | 0.99 | 83.2 | No | | 1960s | |
| SRR2222335 | H(AW) | Penin | Rh mac | 0 | *Penin.* | 23371486 | 96.1 | 1 | 89.6 | No | | 1960s | |
| SRR2225467 | Malayan | Penin. | Rh mac | 10.7 | *Penin.* | 19455031 | 79.3 | 1 | 60.9 | No | | 1960s | |
| SRR2225571 | MR4-H | Penin. | Rh mac | 0.6 | *Penin.* | 22264926 | 74.6 | 0.98 | 64.0 | No | | 1960s | |
| SRR2225573 | Philipp. | Penin. | Rh mac | 0.8 | *Penin.* | 25538996 | 95.2 | 0.99 | 94.6 | No | | 1960s | |
| SRR3135172 | YH1 | Penin. | Rh mac | 5.6 | *Penin.* | 21164226 | 90.1 | 1 | 74.6 | No | | 1960s | |

* Multiplicity of infection (MOI) is % of genome presenting multiplicity of infection; **Group established by whole Genome PCA: *Mf M. fascicularis, Mn M. nemestrina, Penin. Peninsular;* Rh mac Rhesus macaque, *** evidence of genetic exchange (Ex∆)
