# Supplementary figures and images for: Analysis of nuclear and organellar genomes of Plasmodium knowlesi in humans reveals ancient population structure and recent recombination among host-specific subpopulations
Source: PLoS Genet. 2017 Sep 18;13(9):e1007008. doi: 10.1371/journal.pgen.1007008 (PMC5619863; doi:10.1371/journal.pgen.1007008)

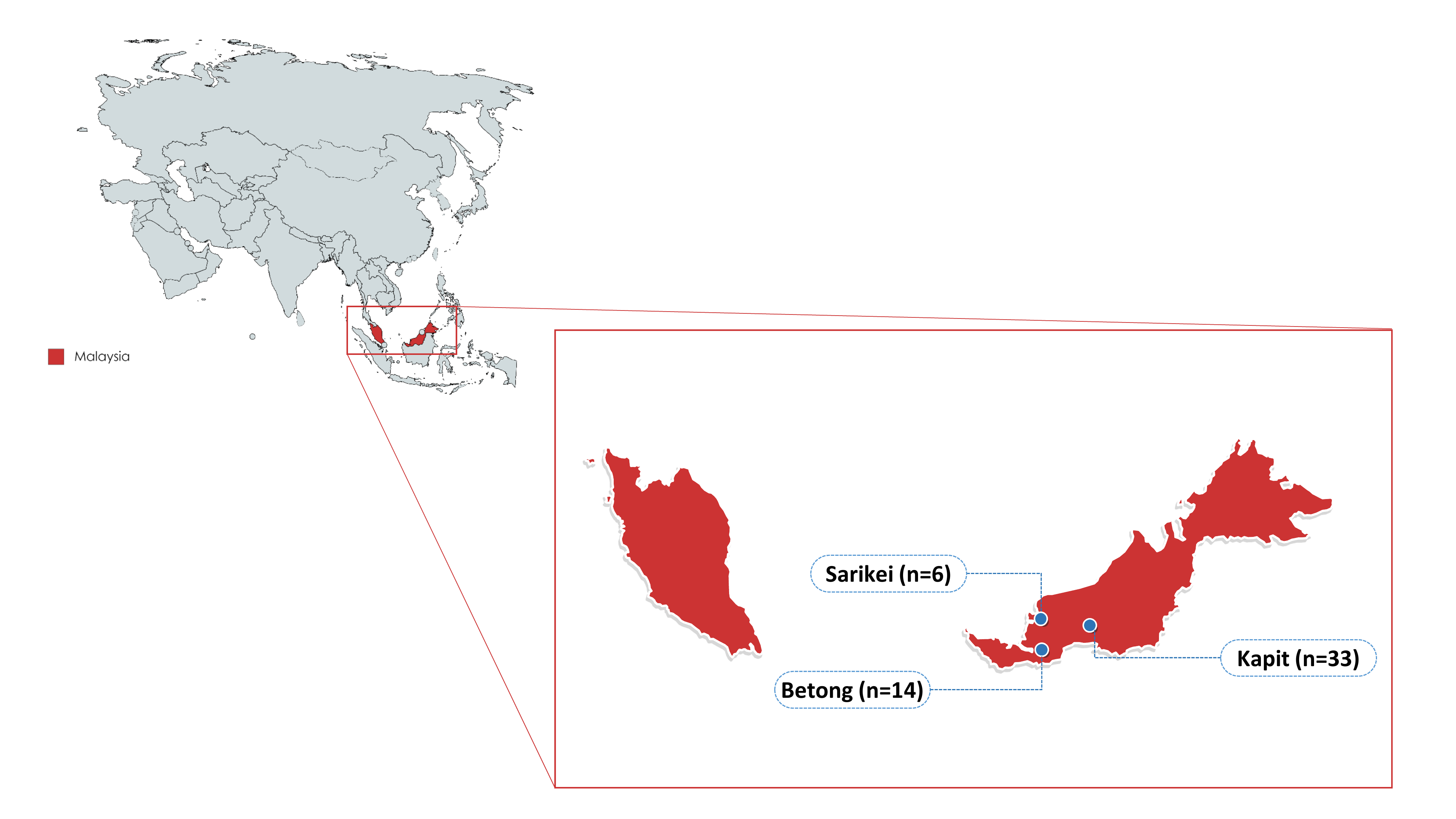

Supplement: S1 Fig — (TIFF) [file pgen.1007008.s003.tiff]

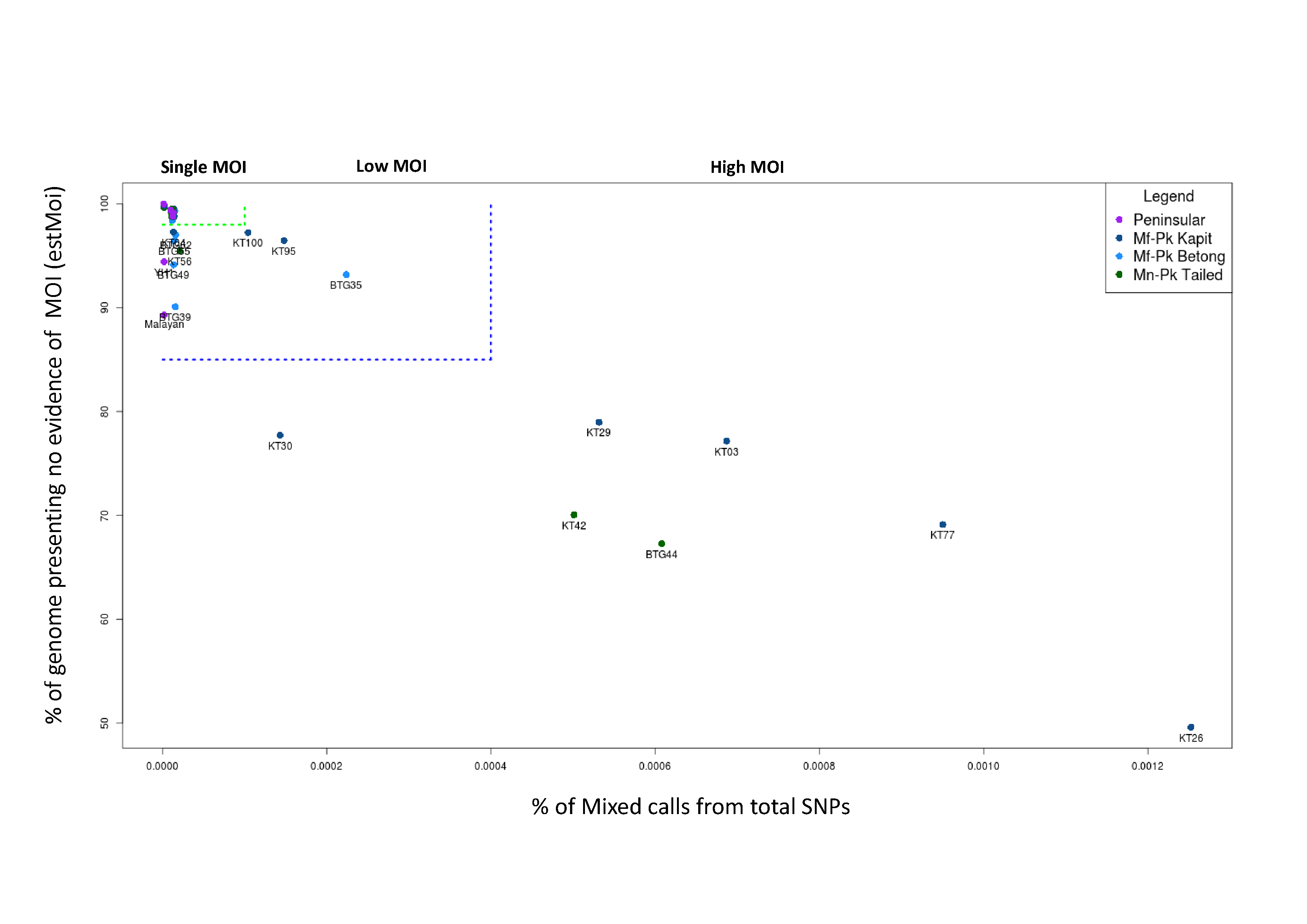

Supplement: S2 Fig — (TIFF) [file pgen.1007008.s004.tiff]

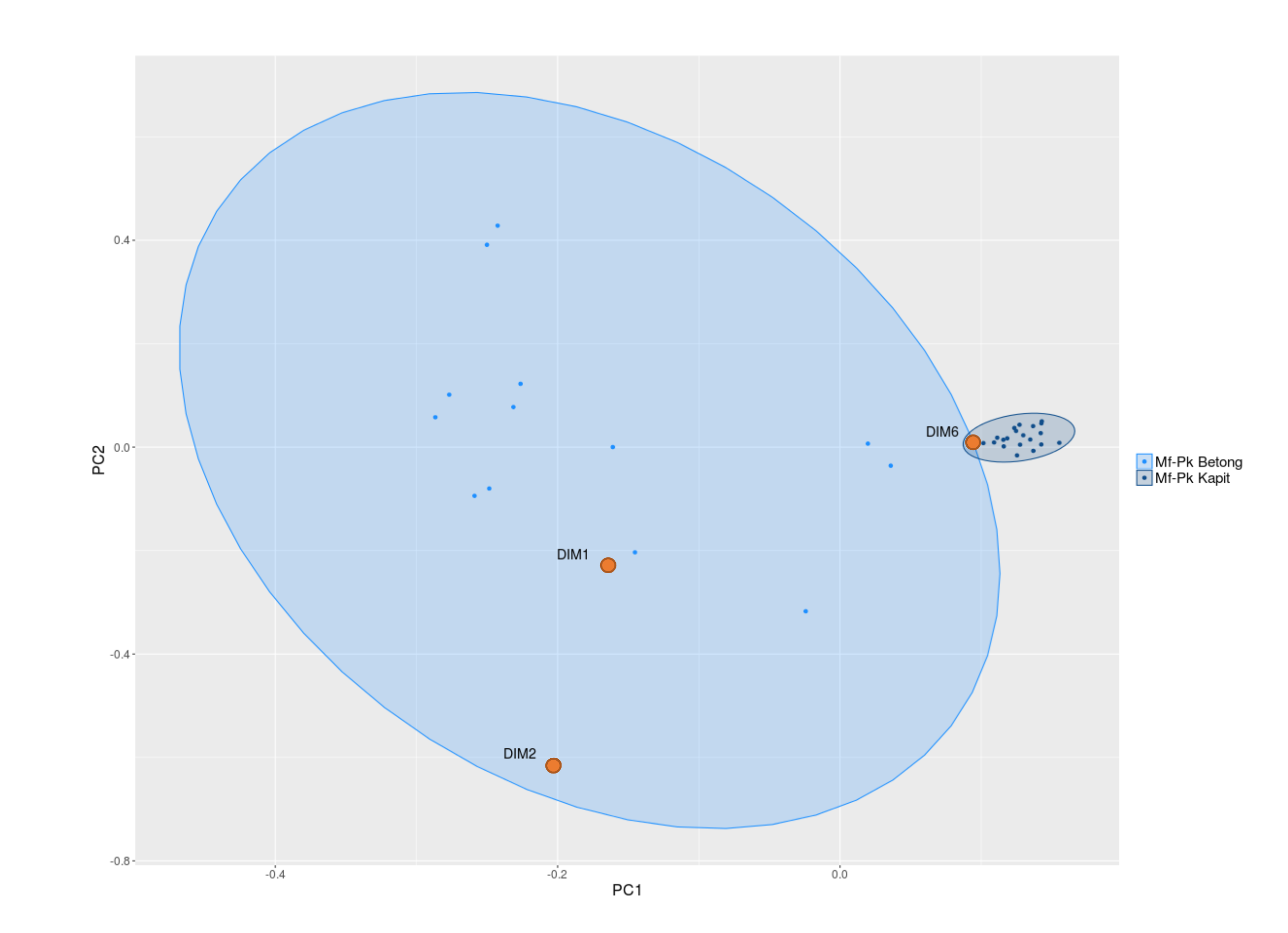

Supplement: S3 Fig — The Mf-Pk Sarikei samples (DIM code in orange) cluster with either one of the two groups, which is consistent with the geographic location of Sarikei as an equidistant region between Kapit and Betong. There is increased diversity of Betong samples compared to the Kapit samples. (TIFF) [file pgen.1007008.s005.tiff]

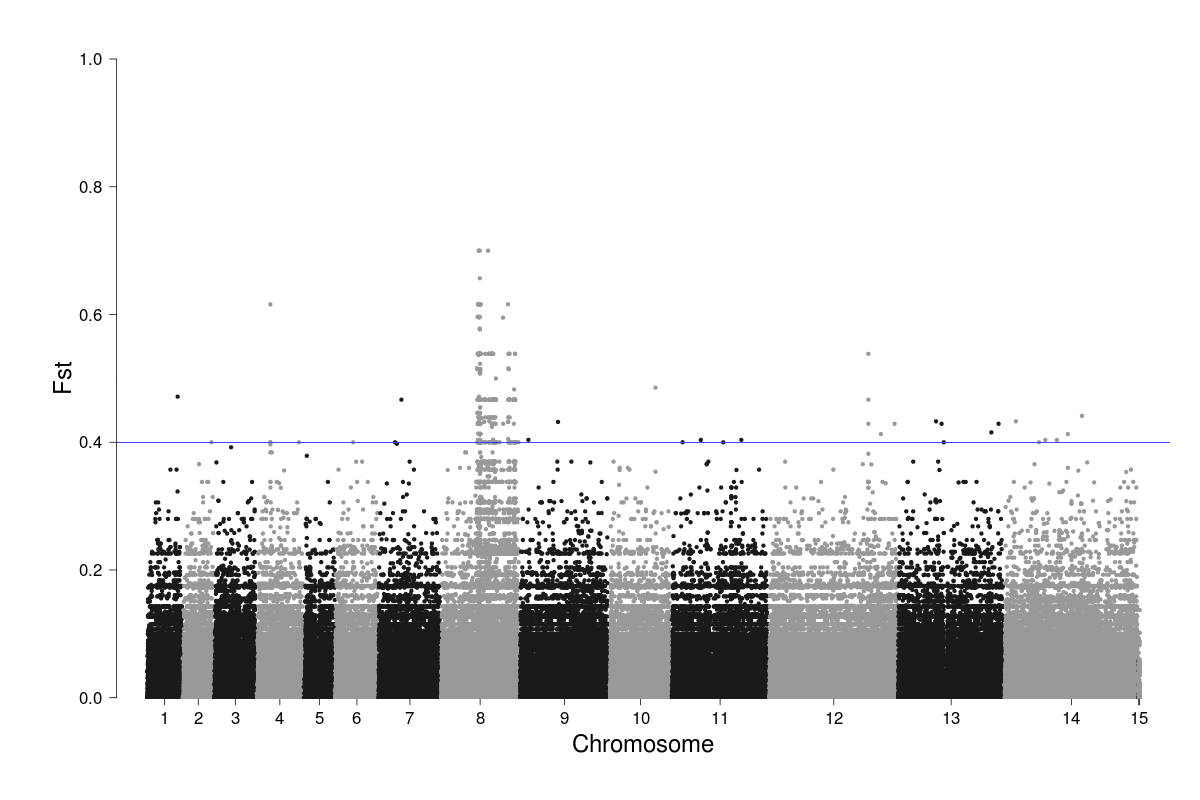

Supplement: S4 Fig — The comparison shows clear abnormalities in several genomic regions in chromosome 8 shown to be a result of genetic exchange with the Mn-Pk genotype. (TIFF) [file pgen.1007008.s006.tiff]

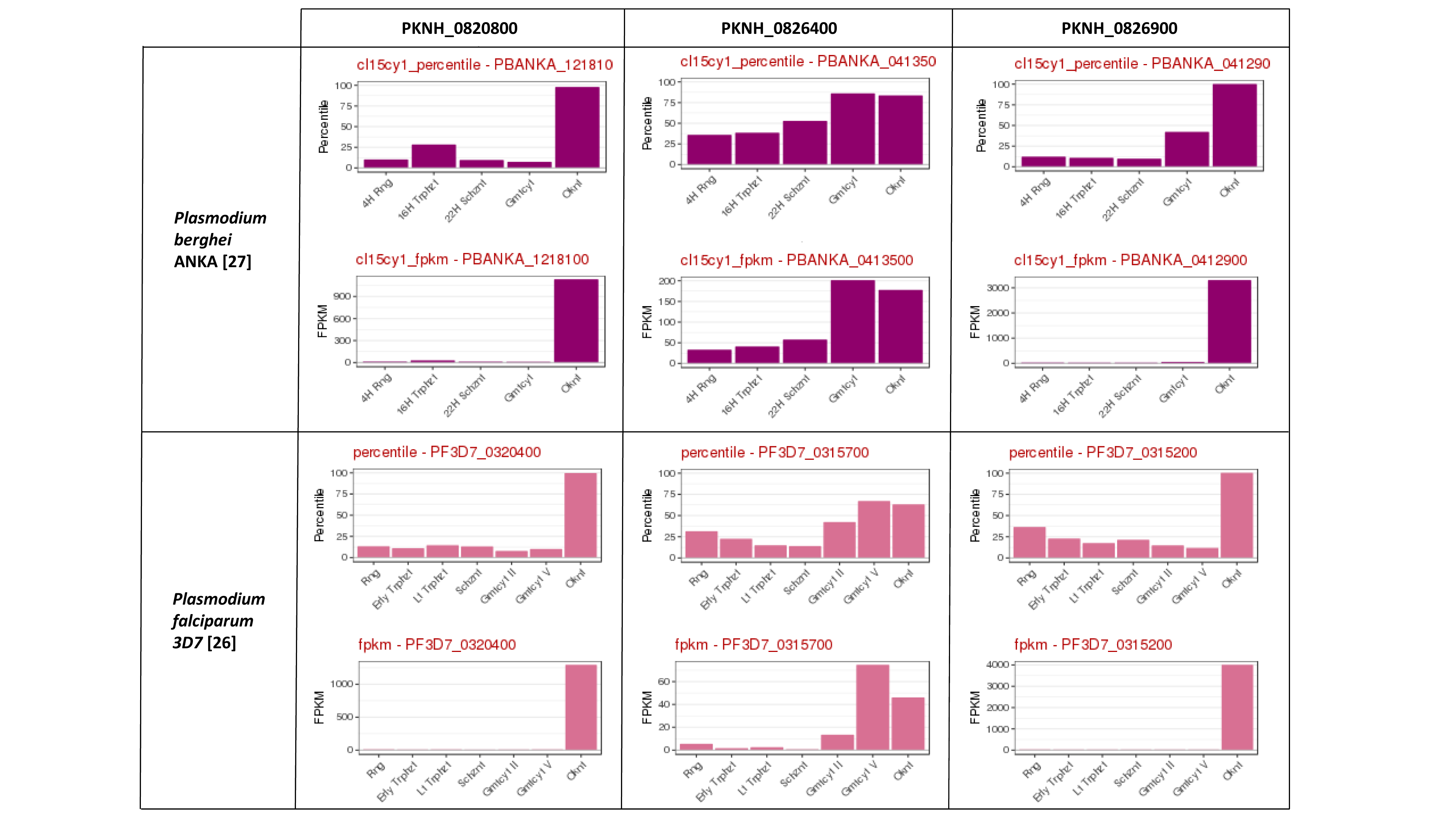

Supplement: S5 Fig — The transcriptomic profiles of the orthologues in P. falciparum [26] and P. berghei [27] for the three genes found to be under strong selection pressure were extracted from PlasmoDB (http://plasmodb.org/plasmo/), including the percentile and the Fragments Per Kilobase of transcript per Million mapped reads (FPKM) plots. These included data for 5 P. berghei stages (4-hour Ring, 16-hour Trophozoite, 22-hour Schizont, Gametocyte and Ookinete) and 7 P. falciparum stages (Ring, early Trophozoite, late Trophozoite, Schizont, Gametocyte stage II, Gametocyte stage V and Ookinete), and showing a clear increased expression in mosquito related stages, particularly the ookinete stage. (TIFF) [file pgen.1007008.s007.tiff]

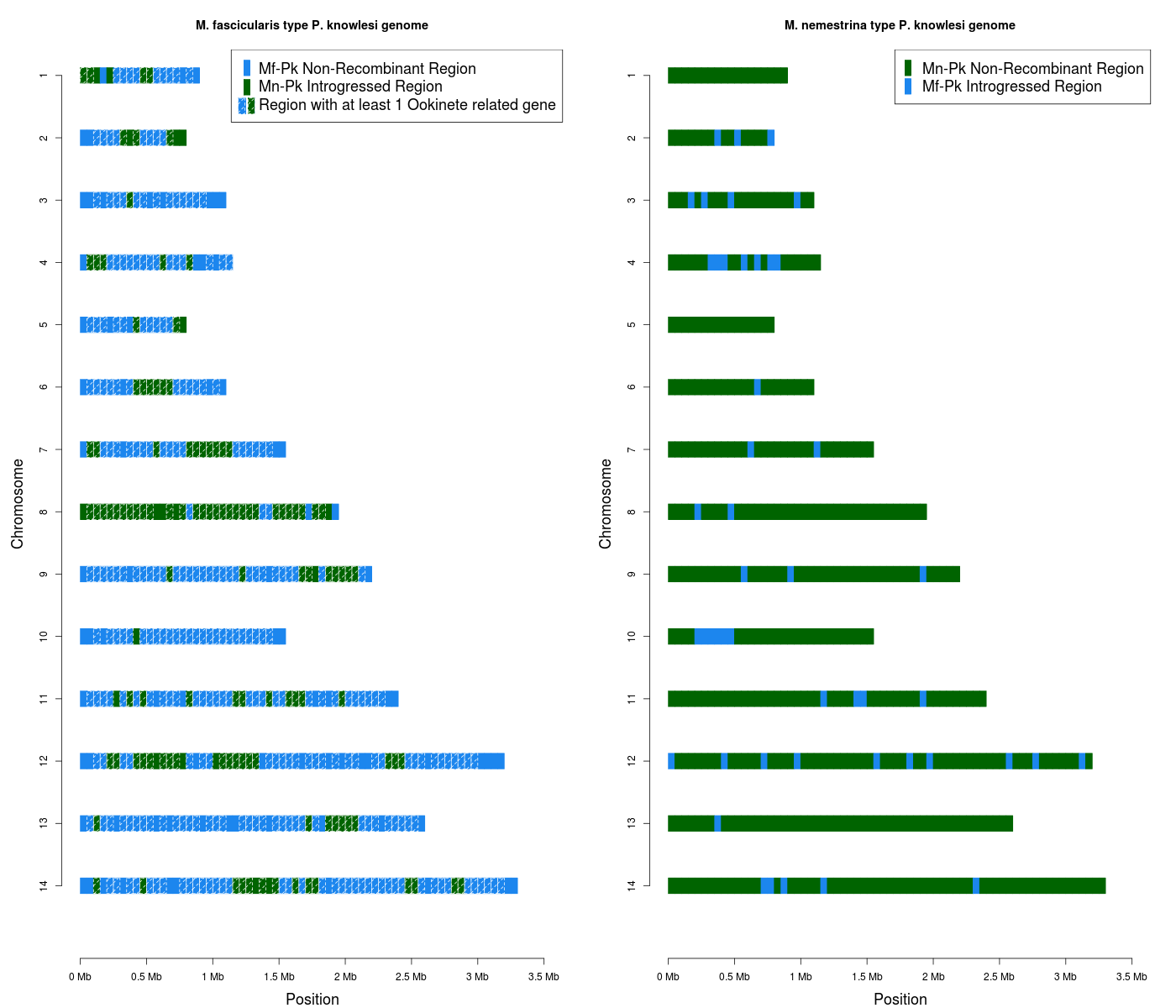

Supplement: S6 Fig — (Left panel) location of introgressions from M. nemestrina P. knowlesi (Mn-Pk) genotype into M. fasciscularis P. knowlesi (Mf-Pk) genotypes, a dashed shaded region has been added where at least 1 gene related with the ookinete life stage of the parasite has been identified based on gene expression for the orthologue genes in P. berghei and/or P. falciparum. (Right panel) location of introgressions from Mf-Pk genotype into Mn-Pk genotypes. (TIFF) [file pgen.1007008.s008.tiff]

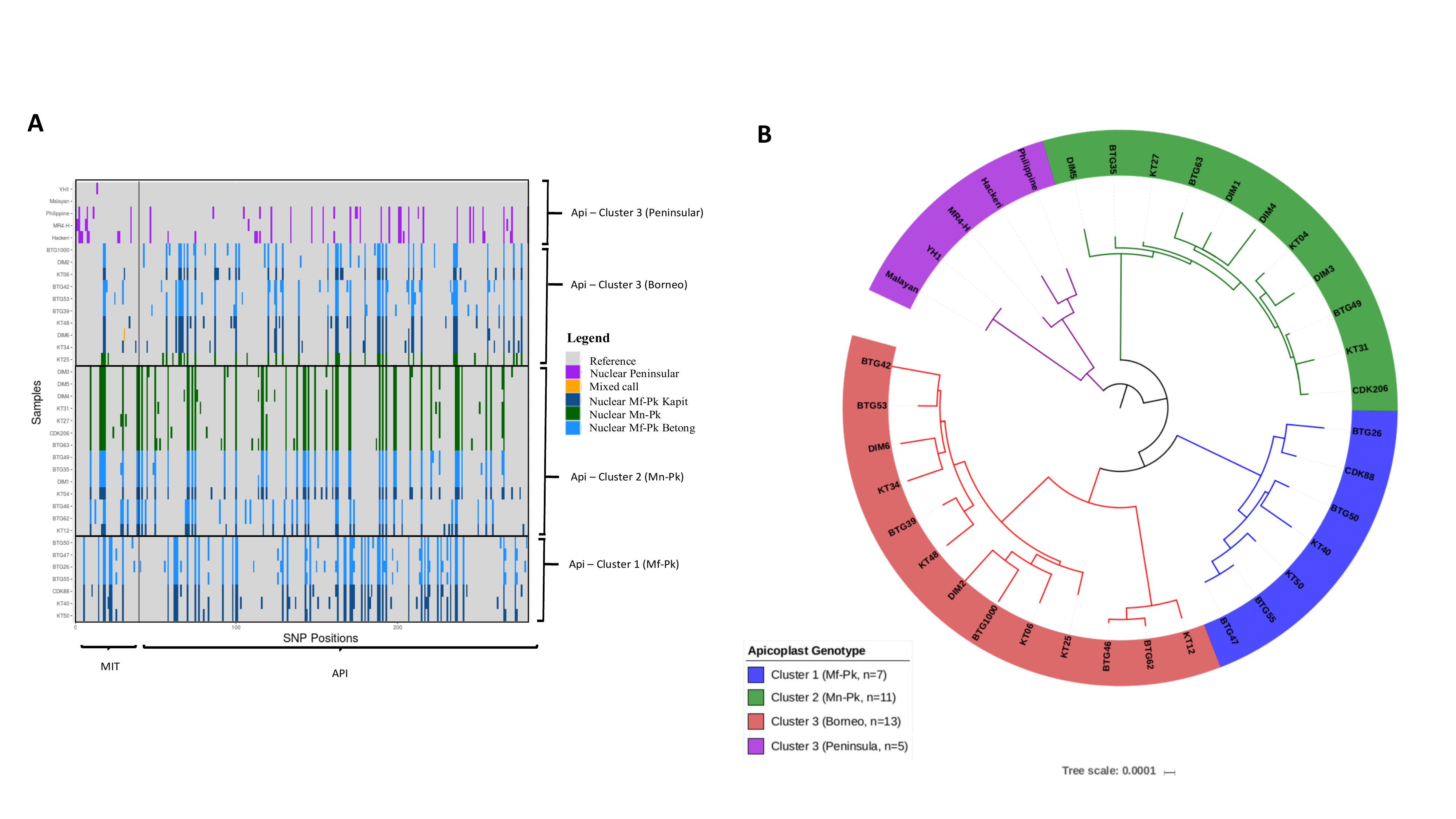

Supplement: S7 Fig — Analysis of organellar mitochondria (MIT) and apicoplast (Api) SNPs confirms clustering into three core haplotype groups a) Haplotype plot for the 36 samples with sufficient coverage across the organellar genomes. Three clearly defined clusters are present. The first cluster represents the mitochondrial genotype found in the Peninsular strains (purple, n = 5) and a set of 10 samples with a highly related haplotype with the smallest inter-cluster average FST (average FST = 0.16) from Borneo Malaysia (represented in red in Fig 2). The second cluster (green in Fig 2) includes the majority of M. nemestrina P. knowlesi (Mn-Pk) nuclear genotype isolates. The third cluster (blue in Fig 2) consists only of isolates with Mf-Pk nuclear genotypes. The presence of samples in the other two clusters with mismatched nuclear and organellar genomes indicates that these two subpopulations have undergone genetic exchange. b) Phylogenetic tree generated using 362 apicoplast SNPs. The tree shows a very similar pattern of clustering to Fig 2. (TIFF) [file pgen.1007008.s009.tiff]
